# Coexistence of primary adenocarcinoma of the lung and Tsukamurella infection: a case report and review of the literature

**DOI:** 10.1186/1752-1947-2-207

**Published:** 2008-06-14

**Authors:** Vinicio A de Jesus Perez, Jeffrey Swigris, Stephen J Ruoss

**Affiliations:** 1 Department of Pulmonary and Critical Care Medicine, Stanford University Medical Center, Pasteur Drive, Stanford, CA 94305, USA; 2 National Jewish Medical and Research Center, Jackson Street, Denver, CO 80206, USA

## Abstract

**Introduction:**

A major diagnostic challenge in the evaluation of a cavitary lung lesion is to distinguish between infectious and malignant etiologies.

**Case presentation:**

We present the case of an elderly man presenting with fever, hemoptysis and a left upper lobe cavitary lesion. Serial sputum cultures grew *Tsukamurella pulmonis*, a rare pathogen associated with cavitary pneumonia in immunocompromised patients. However, despite clinical improvement with antibiotic therapy targeted to the organism, concomitant discovery of a papillary thyroid carcinoma led to a needle biopsy of the cavitary lesion, which showed evidence of primary lung adenocarcinoma.

**Conclusion:**

This is the first description of *Tsukamurella *infection in the setting of primary lung carcinoma. The report also illustrates the potential complex nature of cavitary lesions and emphasizes the need to consider the coexistence of malignant and infectious processes in all patients, especially those with risk factors for malignancy that fail to improve on antibiotic therapy.

## Introduction

A major diagnostic challenge in the evaluation of a cavitary lung lesion is to determine whether it represents an infectious or malignant process. In the majority of cases, it is difficult to distinguish between these two diagnoses with clinical and radiographic data alone, and more invasive testing is usually required to reach a definitive diagnosis. In this report, we describe our experience with a case of cavitary pneumonia resulting from the coexistence of two distinct pathological processes.

## Case presentation

**Figure 1:**
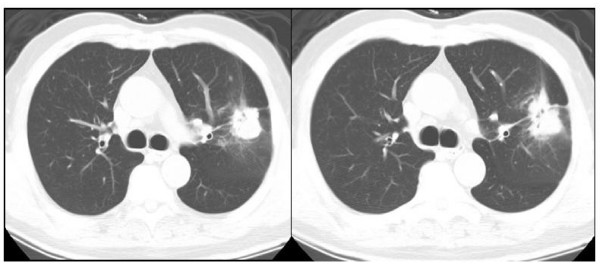
**Computed tomography chest scan at initial visit**. Lung and mediastinal windows show cavitary mass involving both left upper and lower lobes across the major fissure. A calcified ipsilateral lymph node can be seen in the mediastinal windows. This lesion and various lymph nodes were subsequently shown to be positive by positron emission tomography.

A 71-year-old Chinese man who was previously healthy presented to our clinic with a 3-month history of episodic cough with the production of thick yellow sputum. This was accompanied by generalized fatigue, subjective fevers, weight loss and night sweats. Symptoms improved somewhat with over-the-counter antipyretics and cough suppressants. One month prior to the visit, he noticed streaks of bright red hemoptysis, which prompted him to seek medical care. Shortly after an initial chest radiograph (CXR) showed a left upper lobe cavity, a finding later confirmed by computed tomography (CT) scan (Figure 1), he was referred to the chest clinic for further evaluation.

The patient was born and raised in China, where he had lived and worked as a veterinarian for most of his life until he moved to the US in 1981. He had a negative purified protein derivative upon arrival to the US. He had no prior medical problems and was not taking any medication at the time of his visit. He denied any history of alcohol, tobacco or recreational drug use and had no knowledge of sick contacts. On physical examination, he appeared younger than his stated age and in no apparent distress. His examination was relevant only for bronchial breath sounds over the left upper hemithorax.

His CXR and CT scan showed a well-defined left upper lobe cavitary lesion with associated contralateral mediastinal lymphadenopathy. Due to the suspicious appearance of the lesion, a positron emission tomography (PET) scan was ordered along with induced sputum for cultures. Diagnostic bronchoscopy or percutaneous needle biopsy were discussed with the patient and his family, but he did not want any invasive tests. The PET scan showed increased metabolic activity in the left upper lobe lesion as well as in areas of lymph nodes in the contralateral mediastinum; in addition, an area in the left lobe of the thyroid also showed a strong signal which prompted a referral to the thyroid clinic.

During this time, the first sputum sample grew an acid-fast bacillus that was also present in the two subsequent samples. While the initial suspicion was for *Mycobacterium tuberculosis *(TB) or a non-tuberculous *Mycobacterium*, biochemical studies identified the bacteria as *Tsukamurella pulmonis*. Given that infections with this organism can manifest as cavitary pneumonia, and since the patient remained symptomatic, we decided to start him on oral Rifabutin 300 mg daily and oral Levofloxacin 500 mg daily, a regimen chosen based on the antibiotic susceptibility profile (the organism was resistant only to sulfas and tetracycline) and available clinical studies in immunosuppressed patients [1].

After starting therapy, he noticed significant clinical improvement, reduction in sputum volume and resolution of hemoptysis. Upon the recommendation of the endocrine specialist, an aspiration biopsy of the thyroid was performed, which revealed papillary thyroid carcinoma. Given our continued concern regarding the pulmonary lesion and the lack of radiographic improvement after 6 weeks of antibiotic therapy, the patient was again asked and eventually agreed to undergo a percutaneous CT-guided biopsy of the left upper lobe lesion. This revealed adenocarcinoma consistent with a primary lung origin and associated tissue necrosis without evidence of infection. While tissue culture grew *Tsukamurella*, the organism was not identified in tissue sections or in acid-fast stains of tissue sections in which inflammatory changes were absent. Although subsequent staging suggested that it was amenable to surgical resection, the patient opted for medical management while continuing treatment for *Tsukamurella *infection for a total of 6 months. One year after his last cycle of chemotherapy, the patient remains in remission and sputum samples obtained every 3 months after termination of antibiotic therapy have not shown recurrence of *Tsukamurella*.

Originally described as a human pathogen in 1982 [2], members of the genus *Tsukamurell*a belong to the aerobic actinomycetes and are phylogenetically related to species of the *Rhodococus*, *Mycobacterium *and *Nocardia *genera. Morphologically, *Tsukamurella *is a rod-shaped, Gram-positive organism that in most cases demonstrates mild acid-fast staining; more rarely, it may exhibit more substantial acid-fast staining similar to that seen with the *Mycobacterium *species. In culture, growth of *Tsukamurella *requires incubation for 48 hours in aerobic conditions and temperatures between 24 and 37°C. When seeded in a Lowenstein-Jensen agar, *Tsukamurella *colonies exhibit a rough, creamy appearance and, microscopically, these organisms tend to become arranged either in chains or dense clusters.

The presentation of pulmonary infections with Tsukamurella bears a striking similarity to the clinical syndrome seen with mycobacterial infections [2,3]. Clinically, patients may complain of persistent fever, weight loss, anorexia, productive cough and hemoptysis. Radiographic evidence of upper lobe infiltrates is not uncommon and, in the absence of therapy, these may progress to tissue necrosis and cavitation. Immunosuppressed patients may present initially with cavitary lesions, suggesting a more accelerated course in these individuals [1].

Since the original description of Tsukamurella infection occurring in the setting of cavitary pneumonia in a patient who failed traditional tuberculosis therapy, reports of other clinical syndromes have been described, including sepsis [3], catheter-related infections [4], conjunctivitis [5] and infections related to a foreign body [6], among others. Sputum samples may show the presence of Gram-positive rods, but the intensity of acid-fast staining is variable, often leading to confusion with Mycobacteria or Nocardia. To facilitate identification of Tsukamurella, several microbiological tests can be performed (Table 1) [2,7]. The importance of making an accurate microbiological diagnosis is underscored by the fact that Tsukamurella is resistant to many of the drugs used in the treatment of TB or non-tuberculous Mycobacteria, such as streptomycin, cycloserine, rifampin, isoniazid, ethambuthol, p-amino salicylic acid and capreomycin among others [7]. Thus, a delay in diagnosis, or inadequate treatment, may promote progression to cavitary disease and the risk of life-threatening complications, such as massive hemoptysis and respiratory compromise.

**Table 1:** Useful microbiological tests to aid in differentiation of *Tsukamurella *from other bacteria causing cavitary pneumonia

	Mycelium formation	β-galactosidase	Mitomycin C resistance	Para-aminosalicylic acid degradation	Aryl sulphatase	Galactose (carbon source)
*Tsukamurella pulmonis*	**-**	**+**	**+**	**-**	**-**	**+**
*Rhodococcus (R. equi, R. terrae, R. rhodochrous)*	**-**	**-**	**-**	**-**	**-**	**-**
*Mycobacterium (M. chelonei, M. abscessus, M. fortuitum)*	**-**	**+**	**+**	**+**	**-**	**-**
*Nocardia (N. asteroides, N. brasiliensis, N. fracinica)*	**+**	**+**	**+**	**-**	**+**	**-**

Note that *Tsukamurella pulmonis *is the only microorganism that uses galactose as a carbon source. Adapted from Tsukamura and Kawakami [2]

While we failed to observe the physical presence of the organism on the tissue biopsy, the organism grew from the tissue cultures suggesting that absence of organisms may have been due to low bacterial load and/or sampling error. To the best of the authors' knowledge, this is the first report of the coexistence of primary lung adenocarcinoma and *Tsukamurella *in humans. Despite the lack of reports of such an association, several investigators have reported a similar event in patients with TB. Given the limited clinical experience with *Tsukamurella *infections, treatment guidelines have not been well established. The choice of antibiotics is hampered because many of the antibiotics used to treat TB or non-tuberculous *Mycobacteria *are ineffective for *Tsukamurella*. A common approach to treating cavitary pneumonia due to *Tsukamurella *in immunosuppressed patients includes the use of Rifabutin and a fluoroquinolone for 6 to 9 months, with follow-up sputum cultures to document bacterial clearance [1]. It may be useful to perform susceptibility studies in vitro, however, there are no interpretative breakpoints for the genus *Tsukamurella*. Whether other classes of antibiotics may be equally effective either as single therapy or in combination is unclear at this time. Despite the limited evidence in support of our management strategy, it seems to have been effective in controlling the patient's infection as no evidence of recurrent growth was obtained after the treatment period was over. However, future studies should aim at establishing better guidelines to aid in the effective management of this infection.

## Conclusion

This is the first report of *Tsukamurella *pneumonia associated with primary lung carcinoma. Although *Tsukamurella *is a rare cause of cavitary pneumonia that affects mainly immunosuppressed and elderly patients, it should be considered in patients failing to respond to traditional antimicrobial or antituberculous therapy. Clinicians should evaluate patients for malignancy if they fail to respond to appropriate antimicrobial therapy.

## Abbreviations

CT: computed tomography; CXR: chest X-ray; PET: positron emission tomography; TB: tuberculosis

## Competing interests

The authors declare that they have no competing interests.

## Authors' contributions

VAJP compiled the case history, obtained the patient's written consent, drafted the report and performed the review of the literature, JS and SJR were involved in the management of the patient and provided critical input and mentorship during the writing of the report. All authors gave their approval for the final draft of this report.

## Consent

Written informed consent was obtained from the patient for publication of this case report and accompanying images. A copy of the written consent is available for review by the Editor-in-Chief of this journal.
